# The C terminus of p73 is essential for hippocampal development

**DOI:** 10.1073/pnas.2000917117

**Published:** 2020-06-22

**Authors:** Ivano Amelio, Emanuele Panatta, Maria Victoria Niklison-Chirou, Joern R. Steinert, Massimiliano Agostini, Nobuhiro Morone, Richard A. Knight, Gerry Melino

**Affiliations:** ^a^Department of Experimental Medicine, TOR Center, University of Rome Tor Vergata, 00133 Rome, Italy;; ^b^Medical Research Council, Toxicology Unit, University of Cambridge, Cambridge CB2 1QP, United Kingdom;; ^c^School of Life Sciences, University of Nottingham, Nottingham NG7 2RD, United Kingdom;; ^d^Blizard Institute, Barts and the London School of Medicine and Dentistry, Queen Mary University of London, London E1 2AT, United Kingdom

**Keywords:** p53 family, neurodevelopment, alternative splicing

## Abstract

The p53 family member p73 has a complex gene structure, including alternative promoters and alternative splicing of the 3′ UTR. This results in a complex range of isoforms whose biological relevance largely remains to be determined. By deleting exon 13 (which encodes a sterile α motif) from the Trp73 gene, we selectively engineered mice to replace the most abundantly expressed C-terminal isoform, p73α, with a shorter product of alternative splicing, p73β. These mice (*Trp73*^*Δ13/Δ13*^) display severe neurodevelopmental defects with significant functional and morphological abnormalities. Replacement of p73α with p73β results in the depletion of Cajal–Retzius (CR) cells in embryonic stages, thus depriving the developing hippocampus of the pool of neurons necessary for correct hippocampal architecture. Consequently, *Trp73*^*Δ13/Δ13*^ mice display severe hippocampal dysgenesis, reduced synaptic functionality and impaired learning and memory capabilities. Our data shed light on the relevance of p73 alternative splicing and show that the full-length C terminus of p73 is essential for hippocampal development.

## Significance

Alteration of splicing is emerging as a relevant cause of human disease. The C-terminal region of p73 is subject to complex alternative splicing that can give rise to seven different isoforms. Here, using a newly generated mouse model, we determine the functional consequence of in vivo ectopic switch from the physiologically expressed and most abundant isoform p73α to the shorter p73β isoform. Expression of p73β leads to neurodevelopmental defects with functional and morphological abnormalities in the mouse hippocampus. The ectopic isoform switch results in depletion of Cajal–Retzius (CR) neurons in embryonic stages, leading to aberrant hippocampal architecture. Our study indicates that deregulation in p73 alternative splicing might underlie neurodevelopmental human conditions.

p73, a member of the p53 family, has a complex gene organization. Alternative promoters give rise to the N-terminal isoforms TAp73 (which includes the transactivation domain) and ΔNp73 (which is N-terminal truncated); however, a larger complexity emerges at the 3′ UTR of p73 mRNA, where alternative splicing can give rise to seven different isoforms: α, β, γ, σ, ε, ζ, and η (1, 2). p73 plays major roles in development, as genetically modified mice lacking p73 expression (*Trp73*^*−/−*^) display severe neurodevelopmental defects with hippocampal dysgenesis, characterized by unusual arrangements of the cornus ammonis (CA) and dentate gyrus (DG) cell layers (2, 3). Expression of p73 is detected in Cajal–Retzius (CR) cells of developing mouse brain; *Trp73*^*−/−*^ mice display severe depauperation of this pool of neurons and consequent reduced expression of Reelin, the key factor secreted by CR cells to direct the architecture of developing hippocampus (4). The *Trp73*^*−/−*^ postnatal brain displays also severely disrupted architecture of the posterior telencephalon and mild hypoplasia of the rostral cortex, which could also be ascribed to defective CR function (4).

Coexpression of p73 and Reelin is also conserved in fetal human hippocampus. Here p73/Reelin-expressing cells occupy the marginal zone overlying the ammonic and dentate primordia and the cortico-choroid border in the temporal horn ventral cortical hem from 10 gestational weeks (GW). p73/Reelin positivity is also detected from 14 GW in the neuroepithelium near the dentate-fimbrial boundary, while reelin-positive, p73-negative cells are prominent from 14 GW in the prospective strata lacunosum moleculare and radiatum of the cornus ammonis and around midgestation in the dentate molecular layer (ML) and hilus (5). These descriptive data are suggestive of a role of CR (p73/Reelin-positive) cells also in the development of the human brain.

Mice selectively lacking in TAp73 isoforms (*TAp73*^*−/−*^) exhibit a similar phenotype as the *Trp73*^*−/−*^ mice, although less severe (6), while mice lacking the ΔNp73 isoforms show minimal signs of neurodevelopmental impairment (7). The *TAp73*^*−/−*^ mouse model has also been used to help determine the contribution of this group of isoforms to different phenotypes, including male and female fertility, multiciliogenesis, and cancer (6, 8–10), but the individual contributions of the C-terminal isoforms remain elusive.

Cell culture overexpression studies have shown differential transactivation potential for TAp73α compared to that of the shorter isoforms TAp73β and TAp73γ in relation to their proapoptotic transcription targets (11–14). The global genomic binding profile for overexpressed TAp73α and TAp73β by chromatin immunoprecipitation sequencing showed a strong enrichment of AP1 motifs in close proximity to TAp73α−binding sites. Intriguingly, the AP1 motif-containing p73 target genes were found to be selectively up-regulated by TAp73α and down-regulated by TAp73β (15). However, expression profile analysis of C-terminal p73 isoforms shows that p73α is the major isoform physiologically expressed in mouse tissues, with all others below the limit of detection (16), suggesting that this isoform might be biologically relevant.

To address the relevance of p73 3′-alternative splicing, we generated a genetically modified mouse model in which we deleted exon 13 from the *Trp73* gene. The result of this modification is an ectopic switch of expression from p73α to p73β under control of the endogenous promoters. This mouse model (Trp73^Δ13/Δ13^) displays severe morphological and functional neurodevelopmental defects ascribed to the aberrant direction of brain architecture during development. Replacement of p73α with p73β resulted in depletion of the CR cells during embryonic development, resulting in a reduction in Reelin and aberrant hippocampal development (17–22). Thus, our data demonstrate that p73α is essential for correct hippocampal morphogenesis and functionality.

## Results

### Deletion of Exon 13 in the Trp73 Gene Promotes the C-Terminal Isoform Switch from p73α to p73β.

**Fig. 1.**
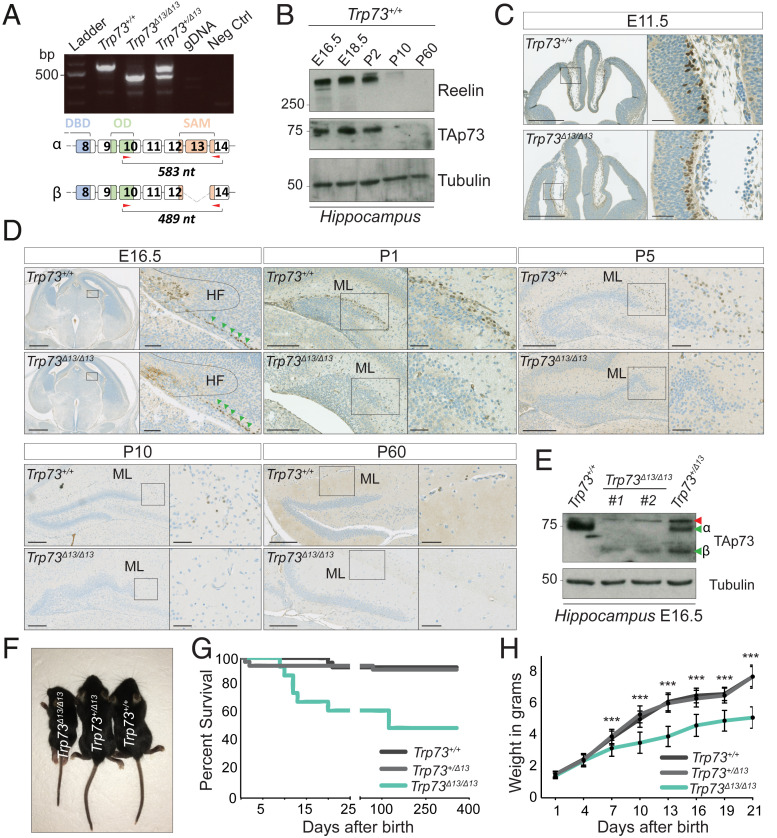
TAp73β replaces TAp73α in the early stages of *Trp73*^*Δ13/Δ13*^ brain development. (*A*, *Top*) RT-PCR was performed to analyze the mouse brain using primer pairs amplifying the C-terminal region (exons 10 to 14) of *Trp73* mRNA. *Trp73*^*+/+*^ expresses only the α isoform (583 nt), *Trp73*^*Δ*1*3/Δ*1*3*^ expresses only the β isoform (489 nt), and *Trp73*^*+/Δ*1*3*^ expresses both isoforms. (*A*, *Bottom*) The α and β C-terminal isoforms. Red arrows indicate the primer- targeted region. DBD, DNA-binding domain; OD, oligo dimerization domain; SAM, sterile α motif domain; gDNA, genomic DNA (from a Trp73^+/+^ mouse ear biopsy). (*B*) Western blot showing TAp73 during hippocampal development. Reelin expression follows a similar pattern. *Trp73*^*+/+*^ hippocampi at different developmental stages (E16.5, E18.5, P2, P10, and P60) were used for protein extraction. β-tubulin served as the loading control. (*C*) IHC performed on mouse brain at embryonic stage E11.5 using an antibody against the transactivation domain of p73 (positive signal in brown). (*Left*) Coronal section of the whole brain in *Trp73*^*+/+*^ (*Top*) and *Trp73*^*Δ13/Δ13*^ (*Bottom*) mice. (Scale bars: 500 µm.) (*Right*) Magnification of the strionuclear neuroepithelium showing TAp73-positive cells. (Scale bars: 50 µm.) (*D*) IHC in mouse brain coronal sections at E16.5, P1, P5, P10, and P60 using an antibody against TAp73 (positive signal in brown). At each embryonic stage, the box in the left images represents a magnification of what is shown in the images on the right. The dotted line indicates the HF, and the green arrows indicate TAp73-positive cells outside of the HF. (Scale bars: At E16.5, 500 µm in the left images and 50 µm in the magnifications; at P1, P5, P10, and P60, 200 µm in the left images and 50 µm in the magnifications.) (*E*) Western blot analysis performed on the mouse hippocampus at E16.5 using an antibody against the transactivation domain of p73. The red arrow indicates a nonspecific product. The green arrows indicate α and β isoforms; #1 and #2 are two different *Trp73*^*Δ13/Δ13*^ mice. β-tubulin served as a loading control. (*F*) *Trp73*^*Δ*1*3/Δ*1*3*^*, Trp73*^*Δ*1*3/+*^, and *Trp73*^*+/+*^ 20-d-old mice (P20). (*G*) Percent survival in *Trp73*^*+/+*^ (*n* = 32), *Trp73*^*+/Δ*1*3*^ (*n* = 40), and *Trp73*^*Δ13/Δ13*^ (*n* = 21) mice. *P* = 0.0012 for *Trp73*^*+/+*^ vs. *Trp73*^*Δ13/Δ13*^; *P* = 0.0019 for *Trp73*^*+/Δ*1*3*^ vs. *Trp73*^*Δ13/Δ13*^; *P* = 0.84 for *Trp73*^*+/+*^ vs. *Trp73*^*+/Δ13*^, log-rank (Mantel–Cox) test. (*H*) Average weight of *Trp73*^*+/+*^ (*n* = 28), *Trp73*^*+/Δ*1*3*^ (*n* = 37), and *Trp73*^*Δ13/Δ13*^ (*n* = 17) mice. Data are presented as mean ± SD. ***P < 0.001 for both *Trp73*^*+/+*^ vs. *Trp73*^*Δ13/Δ13*^ and *Trp73*^*+/Δ*1*3*^ vs. *Trp73*^*Δ13/Δ13*^ at the indicated time points (from 7 to 21 d), two-way ANOVA with Bonferroni’s correction. At each time point (from 1 to 21 d), no significant statistical differences were seen when comparing *Trp73*^*+/+*^ vs. *Trp73*^*+/Δ*1*3*^.

Alternative splicing of the *Trp73* gene can give rise to a wide range of C-terminal isoforms (Fig. 1*A* and *SI Appendix*, Fig. S1*A*), with p73α the most abundantly expressed in the developing hippocampal DG and in layer I of the cortex of the mouse brain (at embryonic day [E] 18.5) (Fig. 1*B* and *SI Appendix*, Fig. S1*B*) (16). Notably, the expression pattern of TAp73 shows stable high protein levels in the developing hippocampus (from E16.5 to postnatal day [P] 2), with quickly declining expression during postnatal development (from P10) (Fig. 1*B*). This highlights the relevance of p73 in hippocampal embryonic and perinatal development.

To determine the role of splicing of the C terminus of p73 during mouse development, we crossed mice carrying *Trp73* floxed alleles with a cytomegalovirus (CMV)-driven CRE recombinase-expressing mouse to specifically delete exon 13 and deplete p73-expressing tissues of the α isoform (*SI Appendix*, Fig. S1*C*). As predicted, in *Trp73*^*Δ13/Δ13*^ mice, the mRNA of the predominant p73 isoform, α, was replaced by p73β, as the deletion of exon 13 results in the generation of an exon 12 to 14 junction, which recapitulates the alternative splicing required to produce p73β (Fig. 1*A* and *SI Appendix*, Fig. S1*A*). Immunohistochemistry (IHC) analysis with a TAp73-selective antibody showed expression of TAp73 in the E11.5 mouse strionuclear neuroepithelium, the early-developing hippocampal and cortical structure, in both *Trp73*^*+/+*^ and *Trp73*^*Δ13/Δ13*^ mice (Fig. 1*C*). However, in subsequent stages of development (from E16.5 onward), including when the DG appears in the hippocampal fissure, expression of TAp73β was strongly reduced in *Trp73*^*Δ13/Δ13*^ mice compared to the levels of TAp73 observed in wild-type mice, and this change was associated with failure of normal DG development (Fig. 1 *D* and *E* and *SI Appendix*, Fig. S1*D*). In particular, the expression pattern of TAp73 was substantially altered. TAp73-positive cells were observed in the hippocampal fissure (HF) of *Trp73*^*+/+*^ mice, but not in that of *Trp73*^*Δ13/Δ13*^ mice (Fig. 1*D*, HF dotted line region); however, *Trp73*^*Δ13/Δ13*^ mice retained a population of TAp73-positive cells outside the HF (Fig. 1*D*, green arrows). After birth, in the ML surrounding the DG, the *Trp73*^*+/+*^ mice had a specific population of TAp73-positive cells that decreased during the postnatal stages of hippocampal development (P1, P5, P10, and P60). In contrast, the developing hippocampus of *Trp73*^*Δ13/Δ13*^ mice from P1 onward is already completely depleted of TAp73-positive cells, reflecting the reduction of TAp73 in the HF at E16.5 (Fig. 1*D*).

At the gross phenotypic level, replacement of p73α with p73β significantly affected the normal growth pattern of mice. Trp73^Δ13/Δ13^ mice were significantly smaller than control mice beginning at P7, and 40% of them died within the first 3 wk of life (Fig. 1 *F*–*H* and *SI Appendix*, Fig. S1 *E* and *F*).

Overall, these data indicate that an artificial constitutive switch of the C-terminal isoforms from α to β impairs gross phenotypic development in mice.

### p73α Is Required for Correct Hippocampal Development.

**Fig. 2.**
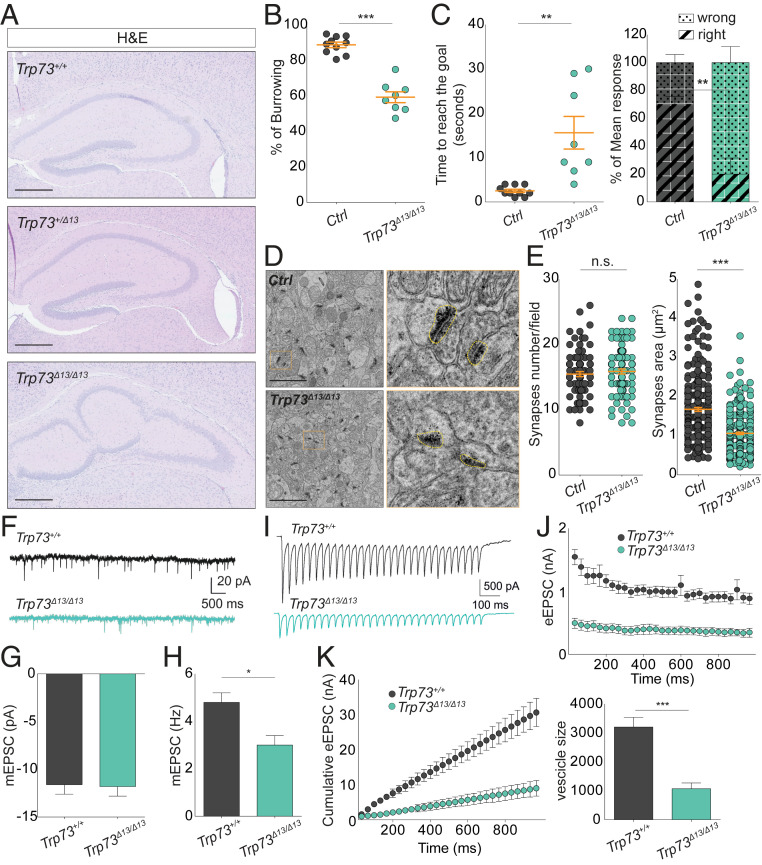
p73α depletion affects mouse behavior by disrupting hippocampal synaptic circuits. (*A*) Hematoxylin and eosin (H&E) staining showing the structure of the hippocampal region in 4-mo-old *Trp73*^*+/+*^, *Trp73*^*+/Δ*1*3*^, and *Trp73*^*Δ13/Δ1*^ mice. (Scale bars: 500 µm.) (*B*) Dot plot showing the burrowing test percentage in control (gray dots; *n* = 5) vs. *Trp73*^*Δ13/Δ13*^ (green dots; *n* = 4) mice. Data are presented as mean ± SEM (orange lines). ***P < 0.0001, unpaired Student’s *t* test. (*C*) T-maze test results. (*Left*) Dot plot showing the time to reach the goal in the same mice used in *B*. Data are presented as mean ± SEM (orange lines). **P = 0.0011, unpaired Student’s *t* test. (*Right*) Histogram showing the mean response percentage (right or wrong) in the same mice used in the left dot plot. Data are presented as mean ± SEM. **P < 0.01, two-way ANOVA with Bonferroni’s correction. In *B* and *C*, a reproducibility test was performed 1 mo later using the same group of mice that were now 3 to 7 mo old. (*D*) TEM photomicrograph analysis of CA1 after sacrificing the mice used in *B* and *C*. Representative TEM images of the synapses (black regions) are shown. (Scale bar: 2 µm.) The images on the right (orange boxes) represent a magnification (32×) of the corresponding images on the left. The yellow dotted lines surround a representative synaptic button. (*E*) Dot plot showing synapse density (*Left*) and area (*Right*) of the mice used in *B* to *D*. A total of 30 electron microscopy pictures/mouse were analyzed using ImageJ. Data are presented as mean ± SEM (orange lines). n.s., not significant. ***P < 0.0001, two-way ANOVA with Bonferroni’s correction. (*F*) Whole-cell patch clamp recordings showing representative spontaneous mEPSCs from *Trp73*^*+/+*^ (*n* = 3) and *Trp73*^*Δ13/Δ13*^ (*n* = 3) mice. (*G*) Amplitudes of mEPSCs were unchanged between genotypes (*n* = 11 neurons for *Trp73*^*+/+*^ and *n* = 19 neurons for *Trp73*^*Δ13/Δ13*^). (*H*) Frequencies (Hz) of mEPSCs were reduced in pyramidal neurons from the same *Trp73*^*+/+*^ and *Trp73*^*Δ13/Δ13*^ neurons used in *G*. Data are presented as mean ± SEM. *P < 0.05, Student’s *t* test. (*I*) Representative eEPSC recordings taken at 30 Hz for 10 s. (*J*) Mean eEPSC amplitudes showing depression within recordings. (*K*) Cumulative eEPSC amplitudes within recordings (*Left*) and back-extrapolation of the linear fits of the last 200 ms of the recording to time point 0 ms revealed an estimate of the available vesicle pool size (*Right*). Data are presented as mean ± SEM. ***P < 0.001, Student’s *t* test.

As p73 shows a specific pattern of expression in the hippocampus, we analyzed hippocampal morphology and functionality to gain a better understanding of the basis of the developmental impairment in the Trp73^Δ13/Δ13^ mice. The hippocampal architecture of *Trp73*^*Δ13/Δ13*^ mice is completely misshapen. Hippocampal neurons were able to form CA and DG cell layers, but these regions were morphologically misassembled and displayed a severely altered architecture (Fig. 2*A*).

To test the functionality of the Trp73^Δ13/Δ13^ hippocampus, we analyzed learning and memory capacities in the genetically modified mice by performing a set of behavioral tests and assessed functionality in electrophysiological studies. The burrowing test showed a significantly reduced (∼30%) capacity for food burrowing in the *Trp73*^*Δ13/Δ13*^ mice compared to the control mice (Fig. 2*B*). We next performed a T-maze test, in which the ability of the mice to reach the goal within the maze was evaluated after 3 consecutive days of training. Both the time to reach the goal and the percentage of correct choices were different in the *Trp73*^*Δ13/Δ13*^ group compared to the control group. Trp73^Δ13/Δ13^ mice showed an increased time to reach the goal (3 s in the control mice vs. 16 s in the Trp73^Δ13/Δ13^ mice) and a reduced frequency of making correct choices (70% correct in the control mice vs. 20% in the *Trp73*^*Δ13/Δ13*^ mice) (Fig. 2*C*). We also analyzed the synapse ultrastructure in the hippocampal CA regions by transmission electron microscopy (TEM). Although the synapse density (number of synapses per area) was similar in the control group and the Trp73^Δ13/Δ13^ group, we observed a significant reduction in synaptic area in the Trp73^Δ13/Δ13^ mice (Fig. 2 *D* and *E*).

We next measured the synaptic activity in CA regions by recording both the miniature excitatory postsynaptic current (mEPSC) and the evoked excitatory postsynaptic current (eEPSC) in acute brain slices. In our analysis of the spontaneous release, we did not observe differences in mEPSC amplitude (readout of postsynaptic function; Fig. 2 *F* and *G*); however, we did observe a significant reduction in mEPSC frequency, from ∼5 Hz in the *Trp73*^*+/+*^ mice to ∼3 Hz in the Trp73^Δ13/Δ13^ mice (Fig. 2*H*), as a readout of presynaptic neurotransmitter release (23, 24). We also evaluated the eEPSC in the same mice by recording the synaptic activity of CA pyramidal neurons following Schaffer collateral stimulation with 300-Hz trains for 900 ms (Fig. 2*I*). Evoked responses were strongly suppressed in mutant hippocampi compared with control hippocampi (Fig. 2*J*). We also estimated the vesicle pool size by linear back-extrapolation of the cumulative eEPSC amplitudes and noted a significant decrease in the size of the available vesicle pool in *Trp73*^*Δ13/Δ13*^ mice compared with the *Trp73*^*+/+*^ controls (Fig. 2*K*), which is consistent with the reduced size of the synaptic area.

These data demonstrate that p73α is required for proper hippocampal development. Expression of an inappropriate p73 C-terminal isoform during mouse development resulted in alteration of physiological hippocampal morphogenesis, with severe disruption of its functionality, memory, and learning and reduction of synaptic transmission. Thus, proper control of p73 alternative splicing is crucial for neurodevelopment.

### Alteration in p73 C-Terminal Isoforms Influences the Postnatal Neuronal Progenitor Pool.

**Fig. 3.**
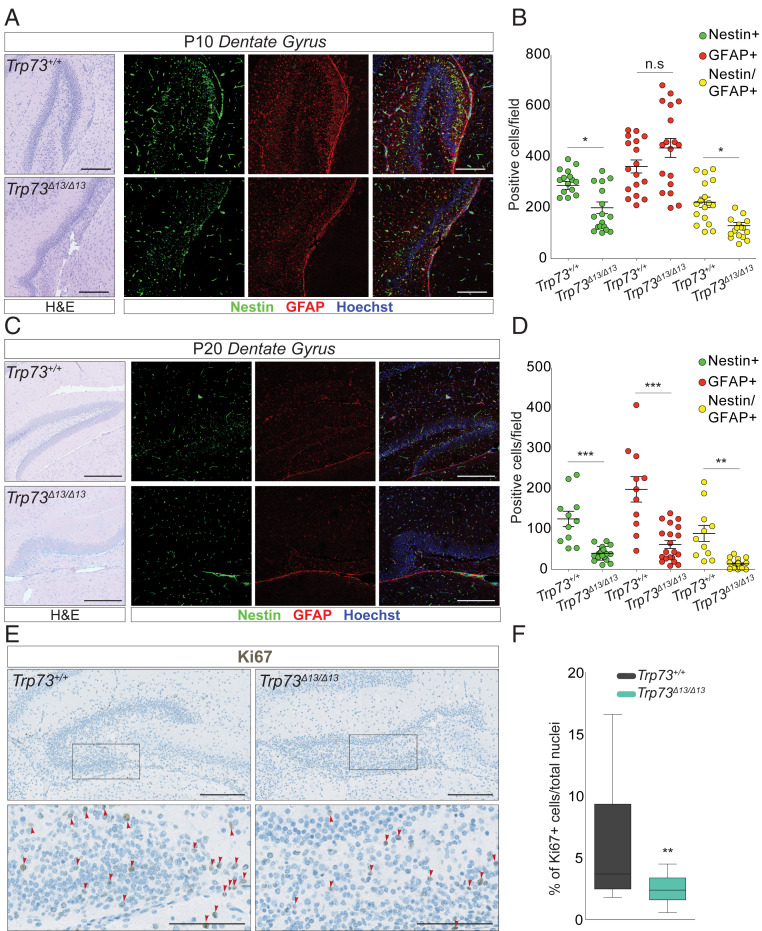
p73α depletion impairs adult neurogenesis in the DG. (*A* and *B*) Neurogenesis analysis performed on 10-d-old mice (P10). Representative IF images from *Trp73*^*+/+*^ (*Top*) and *Trp73*^*Δ13/Δ13*^ (*Bottom*). Nestin (green) and GFAP (red) mark the neuronal precursors in the DG. Hoechst (blue) marks the nuclei. (Scale bars: 200 µm.) (*A*) H&E-stained images showing the morphology of the area used for the IF. (*B*) Dot plot showing the number of Nestin-positive (green), GFAP-positive (red), or double-positive (yellow) cells per field in *Trp73*^*+/+*^ (*n* = 3) and *Trp73*^*Δ13/Δ13*^ (*n* = 3) mice. Data are presented as mean ± SEM. n.s., not significant. *P < 0.05, one-way ANOVA with Bonferroni’s multiple comparison test. (*C* and *D*) Neurogenesis analysis performed on 20-d-old mice (P20). (*C*) Representative IF images from *Trp73*^*+/+*^ (*Top*) and *Trp73*^*Δ13/Δ13*^ (*Bottom*) mice. Nestin (green) and GFAP (red) mark the neuronal precursors in the DG; Hoechst (blue) marks the nuclei. (Scale bar: 400 µm.) H&E images show the morphology of the area used for the IF. (*D*) Dot plot showing the number of Nestin-positive (green), GFAP-positive (red), or double-positive (yellow) cells per field in *Trp73*^*+/+*^ (*n* = 3) and *Trp73*^*Δ13/Δ13*^ (*n* = 3) mice. Data are presented as mean ± SEM. **P < 0.001; ***P < 0.0001, one-way ANOVA with Bonferroni’s multiple comparison test. Volocity software (PerkinElmer) was used to identify Nestin-positive, GFAP-positive, and Nestin/GFAP-positive cells. (*E* and *F*) Ki67 expression analysis by IHC of *Trp73*^*+/+*^ and *Trp73*^*Δ13/Δ13*^ mice at P5 and P10. (*E*) Ki67 representative IHC image from P5 mice. On the bottom (*Left*, *Trp73*^*+/+*^; *Right*, *Trp73*^*Δ13/Δ13*^), the magnified images (scale bars: 100 µm) correspond to the squares in the upper images (scale bars: 200 µm). (*F*) Boxplot showing the percentage of Ki67-positive cells in *Trp73*^*+/+*^ (*n* = 2 at P5; *n* = 3 at P10) and *Trp73*^*Δ13/Δ13*^ (*n* = 2 at P5; *n* = 3 at P10) mice. **P = 0.0011, two-way ANOVA with Bonferroni’s correction. Volocity software (PerkinElmer) was used to count Ki67-positive cells and total nuclei.

During adult neurogenesis, the mature neuronal repertoire is generated from a pool of neuronal progenitors (25, 26). This process primarily involves the subventricular zone and the DG and is essential for developing learning and memory in young mice. In the hippocampus, adult neurogenesis occurs in the granule cell layer of the DG. Here neuronal precursors (GFAP-, Nestin-, and Sox2-positive neurons) differentiate into mature and postmitotic neurons expressing specific differentiation markers (Calbindin, NeuN, and NeuroD) (27). To evaluate the impact of switching from p73α to p73β on the neuronal progenitor pool in the postnatal hippocampus, we analyzed the GFAP- and Nestin-positive progenitor neurons in P10 and P20 mice. As expected, the wild-type mice showed a progressive decline in the neuronal progenitor pool throughout aging (compare Fig. 3 *A* and *B* with Fig. 3 *C* and *D*). Compared to the control mice, mice with a *Trp73*^*Δ13/Δ13*^ genotype displayed a more substantial reduction in Nestin-positive cells at P10 and P20 (Fig. 3 *A*–*D*, green dots), in GFAP-positive cells at P20 only (Fig. 3 *A*–*D*, GFAP^+^, red dots), and in double-positive cells at P10 and P20 (Fig. 3 *A*–*D*, Nestin/GFAP^+^, yellow dots). At P10, *Trp73*^*Δ13/Δ13*^ mice still displayed a significant proportion of neuronal progenitors (160 Nestin/GFAP^+^ cells per field; Fig. 3 *A* and *B*, yellow dots, *Trp73*^*Δ13/Δ13*^), indicating that neurogenesis was still occurring; however, at a later postnatal time point (P20), the number of progenitors was dramatically reduced (15 Nestin/GFAP^+^ cells per field; Fig. 3 *C* and *D*, yellow dots, *Trp73*^*Δ13/Δ13*^). Consistently, there were significantly fewer actively proliferating neurons in *Trp73*^*Δ13/Δ13*^ mice than in their wild-type littermates, as measured by Ki67 positivity in the DG granule cell layer (Fig. 3 *E* and *F*). Thus, this analysis appears compatible with reduced self-renewal capacity in *Trp73*^*Δ13/Δ13*^, which is possibly secondary to the important developmental defects accumulated embryonically and perinatally.

We next analyzed the neurogenesis potential of *Trp73*^*Δ13/Δ13*^ embryogenic precursors. To this end, we measured the ex vivo self-renewal capacity of neurospheres isolated from the E14.5 mouse cortex. We stratified the neurospheres into three populations: small (<25 µm), medium (25 to 50 µm), and large (>50 µm) at passage 0 and small (<20 µm), medium (20 to 40 µm), and large (>40 µm) at passage 1. (The average diameter tends to naturally decrease during passage in culture.) The normal (Gaussian) distribution of the *Trp73*^*+/+*^ neurospheres was progressively different that of the *Trp73*^*Δ13/Δ13*^ samples across the passages. A significantly larger proportion of *Trp73*^*Δ13/Δ13*^ neurospheres was indeed classified as small (<20 µm) compared to that of the wild-type controls (27% vs. 10% at passage 0 and 46% vs. 21% at passage 1) (*SI Appendix*, Fig. S2 *A*–*D*). The number of neurospheres per area also progressively declined in the Trp73^Δ13/Δ13^ samples (*SI Appendix*, Fig. S2*E*). In agreement with these data, Ki67 positivity was reduced in the Trp73^Δ13/Δ13^ cortex (*SI Appendix*, Fig. S2 *F* and *G*). These results are consistent with the previously identified role for p73 during ex vivo neurogenesis in TAp73^−/−^ neurons (28, 29). Overall, a significantly impaired proliferation potential accompanied the morphological defects observed in the *Trp73*^*Δ13/Δ13*^ brain.

### p73α Regulates Function and Distribution of CR Cells.

**Fig. 4.**
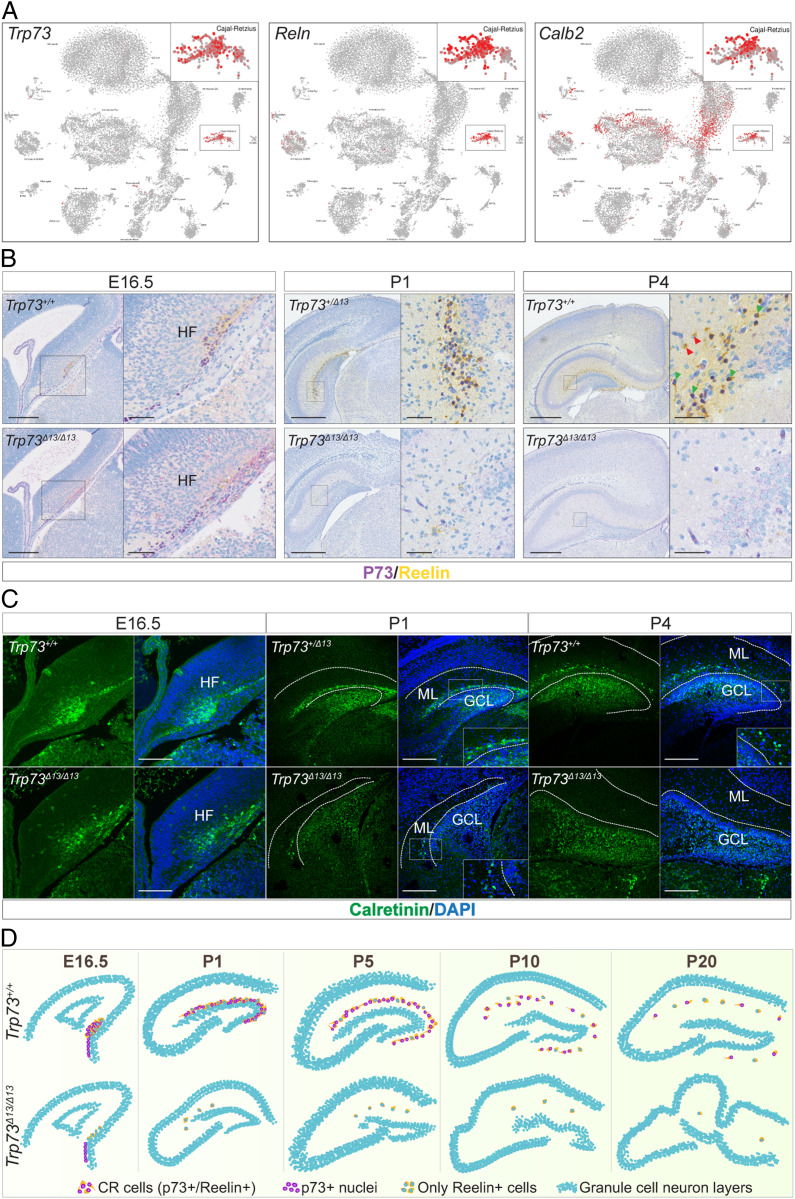
p73α is essential for CR cells during hippocampal development. (*A*) Single-cell RNA sequencing from mouse DG showing that the expression of *Trp73* is almost entirely confined to the CR neurons. *Reln* (Reelin) and *Calb2* (Calbindin 2 or Calretinin) are well-known markers of CR cells. (Data are from ref. 30.) (*B*) IHC colabeling for p73 (purple) and Reelin (yellow) in E16.5 HF (*Left*), P1 (*Middle*), and P4 (*Right*) hippocampi from *Trp73*^*+/+*^ and *Trp73*^*Δ13/Δ13*^ mice. (Scale bars: at E16.5, 200 µm in the left images) and 50 µm in the magnifications; at P1 and P4, 500 µm in the left images and 50 µm in the magnifications. (*C*) Calretinin IF (green) of E16.5 HF (*Left*), P1 DG (*Middle*), and P4 DG (*Right*) from *Trp73*^*+/+*^ and *Trp73*^*Δ13/Δ13*^ mice performed using sections consecutive with those used in *B*. (Scale bars: 200 µm at E16.5, 400 µm at P1, and 500 µm at P4.) DAPI labels the nuclei; white dotted lines delimit the ML. GCL, granule cell layer. (*D*) Graphical representation of hippocampus morphogenesis during development along with the distribution of p73α-positive CR cells.

To determine the mechanism by which p73 participates in brain development, we queried single-cell RNA-sequencing (scRNA-seq) data of the mouse DG during development (30). *Trp73* expression was exclusively observed in CR neurons (Fig. 4*A*). CR cells are a population of Reelin-producing GABAergic neurons present in the developing cerebral cortex and hippocampus that are involved in correct brain organization, allowing cortical stratification and hippocampal development (31). The expression of p73 perfectly matched the expression pattern of the CR markers Reln (Reelin) and Calb2 (Calretinin) (Fig. 4*A*).

To validate these scRNA-seq data, we costained mouse hippocampal regions for TAp73 and Reelin using an IHC multiplex approach. Clear colocalization of TAp73 and Reelin was observed in the HF at E16.5 and in the DG ML at different stages: P1, P4 (Fig. 4*B*), and P10 (*SI Appendix*, Fig. S3*A*). The reduction in TAp73-positive neurons observed in Trp73^Δ13/Δ13^ mice was associated with a consistent reduction in Reelin-expressing cells during all developmental stages (Fig. 4*B* and *SI Appendix*, Figs. S3*A* and S4*C*). Thus, depletion of TAp73 appears to correlate with the depletion of Reelin-producing CR cells. We then tested whether CR cell depletion might be ascribed to a stronger transactivation potential of TAp73β on the proapoptotic p53 family target genes. However, we did not observe any increased apoptosis in the Trp73^Δ13/Δ13^ brain across development (*SI Appendix*, Fig. S4).

To further confirm the absence of CR neurons in *Trp73*^*Δ13/Δ13*^ mice, we also evaluated the expression of an alternative CR marker, Calretinin, by IF at the same developmental stages. Consistently, we found a strong reduction in Calretinin-positive cells specifically in the DG ML during early postnatal development. Notably, Calretinin was expressed in the granule cell layer of the DG in *Trp73*^*+/+*^, *Trp73*^*+/Δ13*^, and *Trp73*^*Δ13/Δ13*^ mice (Fig. 4*C*, P1 and P4; *SI Appendix*, Fig. S3*B*), indicating that its expression is selectively lost in p73-expressing cells of the ML.

Our data indicate that the C-terminal isoform switch from α to β is associated with depletion of the CR cell population. This loss of CR cells leads to severe hippocampal dysgenesis in *Trp73*^*Δ13/Δ13*^ mice. The lack of TAp73β expression in *Trp73*^*Δ13/Δ13*^ mice beginning at E16.5 (Figs. 1 and 4*D*) could also be explained by this depletion of the CR cell population.

## Discussion

The data presented here suggest that p73α is essential for the survival of CR cells and thus for the normal morphological and functional development of the DG. Mice lacking p73α that have the β isoform instead show abnormalities in both the development and function of the DG. In addition, mice lacking exon 13 also have other developmental abnormalities that may be secondary to this primary neurologic deficit.

Correct execution of alternative splicing is crucial for ensuring proper expression of functional proteins. Alteration of this process is indeed emerging as a cause of human disease, and disrupted splicing has the same importance for disease as mutations/alterations in gene structure, which are better characterized (32, 33). In particular, the C-terminal end of p73 can be modified by complex alternative splicing, whose physiological importance is still largely unclear. Here we use a novel genetic approach to demonstrate in vivo that the p73 α isoform is essential for p73 neurodevelopmental function. Our data indicate that the p73 C terminus plays a critical role in CR cell biology, directing proper Reelin-mediated hippocampal development and organization. By removing exon 13 from the *Trp73* gene in mice, we generated a genetic model replacing the most abundant p73 C-terminal isoform, α, with the shorter β isoform. Mice expressing p73β displayed severe hippocampal dysgenesis, reduced learning and memory functions, and a reduced survival expectancy, which largely recapitulated the phenotype of the *Trp73*^*−/−*^ mice completely lacking p73 (2). While the neuronal phenotype of Trp73 has been ascribed to an antiapoptotic function of ΔNp73 (3, 34), here we show that a major role is performed by the TAp73α isoform in the regulation of CR cells during embryonic development.

The underlying molecular mechanism at the basis of the Trp73^Δ13/Δ13^ mouse phenotype remains to be fully clarified. The consequence of the loss of p73α expression vs. the gain of p73β function is a critical unanswered question. The substantial overlap between the Trp73^Δ13/Δ13^ and Trp73^−/−^ phenotypes (hippocampal dysgenesis, associated to early depletion of CR cells, and consequent reduction of Reelin expression) might be suggestive of an important contribution of p73α to the phenotype of our model. This remains speculative, however, and will require formal proof. Remarkably, deletion of exon 13 not only results in an ectopic shift from p73α to p73β, but also influences the mRNA of p73γ and p73ς. Exon 13 in p73γ is part of the 3′ UTR, as it falls after an alternative stop codon generated by the frame shift of the alternative splicing. In p73ς the result of exon 13 deletion should strongly alter the protein structure, producing an aberrant product; however, both isoforms are expressed at significantly lower levels than the protein detection threshold. Thus, a direct contribution of these shorter isoforms to the phenotype is questionable, although this should be confirmed experimentally.

TAp73 is expressed predominantly in CR cells. Its expression pattern during development is indeed consistent with the abundance of the CR population. Our Trp73^Δ13/Δ13^ mice displayed an early depletion of CR neurons, which appears consistent with the early decline in TAp73β expression during development. Thus, correct expression of p73α is required to maintain a proper CR reservoir, which is therefore altered in p73β−expressing mice (*Trp73*^*Δ13/Δ13*^ mice), leading in turn to an early loss of p73-positive cells. Consistent with a critical role for p73 in CR, *Trp73*^*−/−*^ mice also display depletion of CR neurons (2).

Brains of Trp73^Δ13/Δ13^ mice display altered hippocampal synaptic circuitry and postnatal neurogenesis, which influence learning and memory functions in mice. However, our analyses indicate that p73 is expressed exclusively in CR cells, suggesting that p73 does not seem to be directly implicated in maintaining the stem cell characteristics of neuronal precursors. Thus, disruption of proper hippocampal morphogenesis and organization, which appear to be directly regulated by p73 in CR cells, could be causative of a secondary result of inadequate neurogenesis, which further exacerbates the neurologic phenotype of the mice. Trp73^Δ13/Δ13^ mice were also smaller than their wild-type littermates, possibly reflecting their behavioral deficiencies in failing to suckle.

A direct implication of p73 in human neurodevelopmental conditions remains to be assessed. Nonetheless, it is remarkable that the chromosomal region including *TP73* is a target of genetic deletions in such human conditions as the 1p36 deletion syndrome. Interestingly, clinical signs of this syndrome, such as developmental delay, intellectual disability, seizures, hearing loss, and short stature, are highly recapitulated in p73 genetically modified mice (35). The contribution of p73 defects to the spectrum of symptoms in the human syndrome remains unknown, however.

Although our Trp73^Δ13/Δ13^ mice largely recapitulated the phenotype of the Trp73^−/−^ mice, they did not display hydrocephalus. Hydrocephalus in Trp73^−/−^ mice is a result of ventricular enlargement and inappropriate functionality of the ependymal ciliated epithelium (36). Further studies are needed to assess the functionality of ciliated epithelium in *Trp73*^*Δ13/Δ13*^ mice and determine whether p73β can selectively replace p73α in a tissue/region-specific manner.

In conclusion, our newly developed mouse model has proved essential for interrogating the specific role of the C-terminal domains of the p73 protein during neuronal development, providing further molecular insight into the role of *Trp73* during neurogenesis. Our work emphasizes the relevance of p73 in the function and biology of CR cells and leaves open the connection of these mechanisms with human neurodevelopmental conditions.

## Materials and Methods

### Animals.

C57BL/6J mice were housed in the central research facility of the University of Leicester. All experimental work involving animals was approved by the local Ethical Committee and performed in accordance with United Kingdom regulations.

The experiments were performed on sex-matched, age-matched, and strain-matched mice. *Trp73*^*Δ13/Δ13*^ mice were generated using the Cre-LoxP system. First, floxed (Trp73^*fl*/*fl*^) mice were obtained by introducing a vector containing *LoxP* sites flanking exon 13 of the *Trp73* gene, which was replaced by a NeoR cassette to enable selection. Immediately upstream and downstream of these sites, long terminal repeats facilitated incorporation of the vector into embryonic stem cells by homologous recombination. Floxed mice were subsequently crossed with mice ubiquitously expressing Cre-recombinase under the human CMV promoter (CMV-CRE) to delete exon 13 in all tissues.

More details of the study methodology, and primers, sequences, and antibodies (Datasets S1 and S2), are provided in *SI Appendix*.

### Data Availability.

All other relevant data are provided in *SI Appendix* or are available on request.

## Supplementary Material

Supplementary File

Supplementary File

Supplementary File

## Change History

May 5, 2021: The license for this article has been updated.
