# Supplementary material for: The C terminus of p73 is essential for hippocampal development
Source: Proc Natl Acad Sci U S A. 2020 Jun 22;117(27):15694–701. doi: 10.1073/pnas.2000917117 (PMC7355003; doi:10.1073/pnas.2000917117)
Supplement: Supplementary File [file pnas.2000917117.sapp.pdf]

Supporting Information Appendix

The C-terminus of p73 is essential for hippocampal development

Ivano Amelio[1,2,3,@,$], Emanuele Panatta[2,@], Maria Victoria Niklison-Chirou[4], Joern Steinert[2], Agostini Massimiliano[1], Nobuhiro Morone[2], Richard A. Knight[2], Gerry Melino[1,2,$]

[1] Department of Experimental Medicine, TOR Center, University of Rome Tor Vergata, 00133 Rome, Italy.
[2] Medical Research Council, Toxicology Unit, University of Cambridge, Cambridge CB2 1QP, UK.
[3] School of Life Sciences, University of Nottingham, UK
[4] Blizard Institute, Barts and the London School of Medicine and Dentistry, Queen Mary University of London, London E1 2AT, UK

[@] Co-first authors, equally contributed
[$] Corresponding Authors: GM (gm614@mrc-tox.cam.ac.uk) and IA (ivano.amelio@uniroma2.it or ivano.amelio@nottingham.ac.uk) Department of Experimental Medicine, TOR Center, University of Rome Tor Vergata, 00133 Rome, Italy.

Supplementary Material and Methods

Behavioural studies
Burrowing: Mice were housed in the same room (same environmental condition) for 2 hours in cages containing a cylinder filled with 140 gr of food pellets. After 2 hours, the food left in the cylinder (not burrowed) was weighed, and the percentage of burrowing was calculated by subtracting the non-burrowed food weight from the total weight (140 gr). After 1 day of acclimatisation, the experiment was performed for 2 consecutive days; for each day, data were collected. One month later, the same experiment was repeated using the same group of mice. *T-Maze*: The test was conducted following the procedure described by Deacon RM and Rawlins JN (Nat. Protoc. 2006;1(1):7-12). One month later, the same experiment was repeated using the same group of mice. Supporting information and statistical data are detailed in the legend of Fig 2.

Electrophysiology
Brain slices were prepared from animals killed by cervical dislocation. Whole-cell patch recordings were made from visually identified mouse CA1 neurons in acute brain slices (300 µm thick) of the hippocampus as described previously (37). Pasteur pipettes were pulled from glass capillaries (GC150F-7.5, o.d. 1.5 mm, Harvard Apparatus) and had resistances of 3.5–5 MΩ when filled with the pipette solution. Series resistances were between 15 and 20 MΩ (series resistance compensation and prediction were approximately 70%). Data were recorded using a Multiclamp 700B amplifier (Molecular Devices). Stimulation, data acquisition, and analysis were performed using pClamp 10.4 and Clampfit 10.4 (Molecular Devices). The slices were incubated in artificial cerebrospinal fluid (aCSF) to maintain them after preparation, and perfusion was performed during recordings (125 mM NaCl, 2.5 mM KCl, 26 mM NaHCO3, 10 mM glucose, 1.25 mM NaH2PO4, 2 mM sodium pyruvate, 3 mM myo-inositol, 2 mM $CaCl_2$, 1 mM MgCl2, and 0.5 mM ascorbic acid; the pH was 7.4 after it was gassed with 95% O2, 5% CO2). Osmolarity was adjusted to 310 mosmol/l. A low sodium aCSF was used during the preparation of slices, and the composition was the same as that listed above for aCSF, except that NaCl was replaced by 200 mM sucrose, and CaCl2 and MgCl2 were changed to 0.1 mM and 4 mM, respectively. The pipette solution for whole-cell recordings contained 120 mM potassium methanesulfonate, 10 mM HEPES, 0.2 mM EGTA, 4 mM K-ATP, 0.3 mM Na-GTP, 8 mM NaCl, and 10 mM KCl, and was at pH 7.4 with osmolarity ranging from 280 to 290 mosmol/l. Synaptic stimulation at the Schaffer collateral was achieved using an isolated stimulator (Digitimer; 1–10 V, 0.1–0.2 ms) via a bipolar platinum electrode.

www.pnas.org/cgi/doi/10.1073/pnas.2000917117

End-point PCR

Mouse genotyping was performed using a RED Extract-N-Amp^{TM} Tissue PCR kit (Sigma) according to the manufacturer's instructions. The primers used are listed in Table 1. P73 C-terminal isoform-specific PCR was performed starting with brain RNA that was extracted using TRIzol® Reagent (Ambion) according to the manufacturer's instructions. Two micrograms of RNA were reverse transcribed using SuperScript® III Reverse Transcriptase with oligo-dT (Thermo Fisher) according to the manufacturer's instructions. The primer sequences for all *Trp73* C-terminal isoforms are listed in Table 1. Reactions were carried out using the RED Extract-N-Amp^{TM} PCR ReadyMix^{TM} (Sigma). The products were run on a 1-2% agarose gel in TAE buffer and were visualized using a Gene Genius Bio Imaging System (Syngene). The obtained PCR products were sequenced.

Immunofluorescence (IF)

Dissected whole brains were fixed in 4% paraformaldehyde for 48 h and then were embedded in paraffin. Coronal brain sections (5 µm thick) were obtained for analysis of the hippocampus. Sections were deparaffinised by immersion in xylene (Sigma) followed by a gradient of 100%-70% ethanol (Sigma). Antigen retrieval was performed by heating in a microwave at 295 W for 15 minutes in 0.01 M sodium citrate. Tissue was blocked with PBS containing 5% normal goat serum for 1 h at room temperature, and then the tissue was incubated with a primary antibody in PBS containing 5% normal goat serum overnight at 4°C in a humidified atmosphere. See Table 2 for antibody information. Samples were washed 3 times in PBS and incubated for 1 h with Alexa Fluor^{TM} secondary antibodies (Molecular Probes; Invitrogen) at RT. Samples were washed 3× in PBS and mounted using Prolong Gold Antifade mounting media (Thermofisher; P36930). Images were acquired through confocal microscopy (LSM 510, Zeiss). Number of Nestin/GFAP positive cell counting per microscopy field was determined using ImageJ.

Immunohistochemistry (IHC)

Tissue sections were fixed and embedded as described above. IHC was carried out using a Ventana Discovery Ultra platform (Roche). Deparaffinization was performed by immersion in Ventana Discovery Wash buffer (Roche; 950-510) for 3 × 8-minute washes at 69°C. Antigen retrieval was performed by incubation in Ventana CC1 buffer at 95°C for 32 minutes. The slides were blocked by incubation with Discovery goat IgG block (Roche; 760-6008) for 12 min at 37°C. Primary antibody incubations were carried out using EnVision Flex antibody diluent (Dako; DM830). See Table 2 for antibody information. The detection reaction was performed using an UltraMap DAB anti-Rb detection kit (Roche) for single staining of TAp73 or an UltraMap HRP anti-Rb detection kit (Roche) in combination with an UltraMap AP anti-Ms detection kit (Roche) to co-stain TAp73 and Reelin, according to the manufacturer's instructions. H&E staining was performed with the following sequential steps: 2 oven incubations (15 min each), 2 incubations in xylene (2 min each), 3 incubations in 100% IMS (1 min each), wash with water (1 min), incubation in Harri's haematoxylin (15 min), wash with water twice (1 min each), incubation in 1% HCL alcohol (30 secs), wash with water (6 min), wash with water (2 min), incubation in 0.5% eosin (3 min), wash with water (2 min), incubation in 80% IMS (1 min), 3 incubations in 100% IMS (1 min each), and 3 incubations in 3 xylene (1 min each). The images were captured with a NanoZoomer XR digital pathology slide scanner, and images were processed with NDP.view.2.3.1 software (Hamamatsu). Number of Ki67 positive cell counting, normalised on the total number of nuclei, was determined using ImageJ.

***In situ* hybridization (ISH)**

Mouse brains were embedded in frozen specimen medium Killik (Bio-Optica) after an overnight incubation in 4% paraformaldehyde followed by an overnight incubation in 0.5 M sucrose. Thin sections (14 µm) were cut and mounted on Superfrost glass slides. Slides were then fixed for 10 min in 4% paraformaldehyde and were acetylated for 10 min in triethanolamine/acetic anhydride. Slides were then hybridized overnight at 46°C using 30 nM digoxigenin probes for detection (miRCURY LNA; Exiqon). After hybridization, slides were washed (20 min in 5× SSC, two times for 30 min in Tween 20/SSC at 50°C, two times for 15 min in 0.2× SSC and 15 min in PBS at RT). After 1 h of incubation in a blocking solution at RT, slides were hybridized for 2 h with an alkaline phosphatase-conjugated antidigoxigenin Fab fragment (1:200 dilution; Roche) at RT. After two 20 min washes, detection was performed by incubating 250 µl of nitroblue tetrazolium/BCIP (1-STEP; Thermo Fisher Scientific) together with 2 mM levamisole on the slides for 16 h in the dark at RT. Table 1 lists the primers used to generate the ISH probe.

Neurosphere assay
Whole cortexes from E14.5 mice were carefully dissected and immediately stored in cold Dulbecco's phosphate-buffered saline (StemCell Technologies; without Ca^{++} and Mg^{++}) containing 2% glucose. A single cell suspension of cortical neurons was obtained by carefully pipetting the whole cortex (E14.5) in neuronal basal medium supplied with NeuroCult® Stem Cell Proliferation Supplements (StemCell Technologies). For each passage in culture, $5x10^5$ cortical neurons were seeded in 10 mm dishes. Images were acquired with an Axiovert 25 microscope (Zeiss). Neurosphere density and diameter were determined using ImageJ.

Real Time qPCR
RNA was extracted from the hippocampus and cortex using TRIzol® Reagent (Ambion) according to the manufacturer's instructions. The concentration and purity of RNA were measured using a nanodrop spectrophotometer (Thermo Fisher). One microgram of RNA was used for reverse transcription reaction using a RevertAid minus first strand cDNA synthesis kit (Thermo Fisher) according to the manufacturer's instructions. Primer sequences (Sigma) for TAp73 and ΔNp73 are listed in Table 1. Reactions were carried out in triplicate using Fast SYBR Green PCR Master Mix (Thermofisher #4385612). The relative quantification was obtained using an Applied Biosystems 7500 thermocycler, and quantitation was calculated with the comparative ($^{\Delta\Delta}$Ct) method; expression values were normalised to *gapdh*, which was used as an endogenous control.

Ultrathin Section Transmission Electron Microscopy (TEM)
Mouse brains were fixed with 2.5% glutaraldehyde (GA) and 2% paraformaldehyde (PFA) in NaHCa buffer (100 mM NaCl, 30 mM HEPES, and 2 mM CaCl2, adjusted to pH 7.4 with NaOH) for more than a few hours at room temperature. The specimens were then subjected to conventional post-fixation treatment with 0.25% osmium tetroxide/0.25% potassium ferrocyanide and 1% tannic acid. After staining en bloc with 5% aqueous uranyl acetate, dehydration with an ethanol series and infiltration were completed for plastic embedding in TER (TAAB epoxy resin). After the polymerization at 65°C for a few days, ultrathin sections (~60 nm) that were obtained from an Ultramicrotome (Leica Ultracut UCT, Vienna Austria) were mounted in EM grids, stained with lead citrate, and then observed by FEI Talos F200C 200 kV transmission electron microscopy (Thermo Fischer Scientific, Oregon USA) with a Ceta-16M CMOS-based camera (4 k x 4 k pixels under a 16 bit dynamic range).

Western Blot (WB)
Protein extraction was performed on ice. Dissected whole hippocampi were washed in PBS and lysed by homogenization in RIPA buffer (1 M Tris-HCL pH 7.4, 5 M NaCl, 10% sodium deoxycholate, 10% sodium dodecyl sulphate, 1% NP-40, and 0.1 M PMSF) supplemented with a protease inhibitor cocktail (Sigma) and 1 M dithiothreitol (DTT). Debris was pelleted by centrifugation at 10,000 x g for 5 minutes, and the supernatant was collected. Protein concentrations were determined using a BCA assay (Thermo Fisher). Laemmli sample buffer (4x) (BioRad) was added to the protein samples, which was followed by denaturation: heating at 98°C for 10 minutes. Fifteen micrograms of protein was run on an 8% resolving gel (National Diagnostics) at 150 V for 1 h. Protein was transferred to PVDF membranes (Thermo Fisher) in 1x transfer buffer (Thermo Fisher) containing 10% (v/v) methanol by applying 0.8 mA per cm^2 of membrane for 2 h on ice. Membranes were blocked in 5% milk (Marvel) in TBS/0.1% Tween 20 (TBST) for 1 h at RT before incubation with a primary antibody (diluted in 5% milk in TBST) overnight at 4°C. See Table 2 for antibody information. Membranes were washed 3X in TBST and were incubated with a goat anti-mouse/HRP conjugated secondary antibody (Thermofisher #31430) diluted 1:10000 in TBST for 1 h at RT. Membranes were washed 3X in TBST and then were incubated with SuperSignal™ West Dura Extended Duration Substrate (Thermo Fisher) for 2 min before visualisation using radiographic films.

Legends to Supplementary Figures

Supplementary figure 1. (**A**) End point PCR showing the transcriptional expression of *Trp73* C-terminal isoforms in $Trp73^{+/+}$, $Trp73^{\Delta13/\Delta13}$, and $Trp73^{+/\Delta13}$ mice. C-terminal isoform-specific primer pairs were used to amplify β (*beta*), γ (gamma), ζ (zeta), ε (epsilon), and δ (delta). (**B**) RNA *in situ* hybridization (ISH) on mouse brain at E18.5 using a probe targeting *Trp73α*. In the middle, the picture shows a whole brain (coronal section; bar=1 mm). On top, a magnification of the cortical area (bar=250 μm); on bottom, a magnification of the DG (bar=250 μm). (**C**) Genotype strategy by end-point PCR amplification of wild type (P3-P5 primer pair), floxed (P3-P4a primer pair), and exon 13 knockout (P1h-P5 primer pair) alleles. (**D**) Real-time qPCR showing the relative expression of TA and DN *Trp73* isoforms in the hippocampus and cortex of $Trp73^{+/+}$, $Trp73^{\Delta13/\Delta13}$ and $Trp73^{+/\Delta13}$ mice. *Gapdh* was used as a housekeeping gene. (**E**) Gain of weight from P1 to P21 of the mice shown in Fig 1H. n.s.=not significant; **p-value<0.01; unpaired Student's t-tests were used. (**F**) The average weight of the male and female mice shown in Fig. 1H.

Supplementary figure 2. (**A**-**E**) Neurosphere assay analysis performed using E14.5 cortical neurons. (**A**) Representative images of the neurosphere at passage 0; bars=200 μm; (#1 and #2 are two different mice). (**B**) Graph showing the distribution of the $Trp73^{+/+}$ and $Trp73^{\Delta13/\Delta13}$ neurosphere populations at passage 0. (**C**) Representative images of the neurosphere at passage 1; bars=100 μm. (**D**) Graph showing the distribution of the $Trp73^{+/+}$ and $Trp73^{\Delta13/\Delta13}$ neurosphere populations at passage 1. Note that in (B) and (D), the $Trp73^{\Delta13/\Delta13}$ neurosphere distribution diverges from the $Trp73^{+/+}$ Gaussian distribution. The deviation from the Gaussian distribution was calculated with a chi-squared test using 2 degrees of freedom (D.F.); C.L.=confidence levels. (**E**) Histogram showing $Trp73^{+/+}$ and $Trp73^{\Delta13/\Delta13}$ neurosphere density; n.s.=not significant; **p-value<0.01; unpaired t-tests were used. Note that in (B), (D), and (E) 13 images/mouse ($Trp73^{+/+}$ n=4; $Trp73^{\Delta13/\Delta13}$ n=3) were used. (**F**) Representative Ki67 IHC staining of E16.5 mice. The magnifications of the cortical area (bar=300 μm) correspond to the black square in the whole brain images (bar=1 mm). The yellow dotted line surrounds the region used to count Ki67-positive cells. (**G**) Box plot showing the percentage of Ki67 positive cells and total nuclei; 10 images/mouse ($Trp73^{+/+}$ n=2, and $Trp73^{\Delta13/\Delta13}$ n=2) were analysed using Volocity software (PerkinElmer); **p-value<0.01; two-way ANOVA tests with Bonferroni's correction were used.

Supplementary Figure 3. (**A**) IHC colabelling of TAp73 (purple) and Reelin (yellow) in P10 DG. The black boxes are magnifications (40X) of a region of the molecular layer in sections from both $Trp73^{+/+}$ and $Trp73^{\Delta13/\Delta13}$. Red arrows indicate cells that are positive for Reelin but negative for p73; green arrows indicate cells positive for both TAp73 and Reelin; bars=400 μm. (**B**) Calretinin IF (green) on P10 DG. The white dotted lines indicate the molecular layer (ML); the magnification in $Trp73^{+/+}$ section shows Calretinin-positive cells in the ML. $Trp73^{\Delta13/\Delta13}$ mice do not have Calretinin-positive cells in the ML; bars=500 μm. (**C**) WB showing Reelin and Calretinin expression in the same samples shown in Fig. 1C; #1 and #2 are two different $Trp73^{\Delta13/\Delta13}$ mice; β-Tubulin (Tubulin) was used as a loading control.

Supplementary Figure 4. (**A-C**) p73 IHC and TUNEL assay (green) in mouse brains of mice at E11.5 (A), E16.5 (B), P1 (C). No major differences are observed between $Trp73^{+/+}$ and $Trp73^{\Delta13/\Delta13}$. Negative controls have been performed on samples incubated with solution only in absence of enzyme. Bars in A indicate 500 μm, in B and C indicate 50 μm. ML: Molecular Layer.

Supp. Fig1

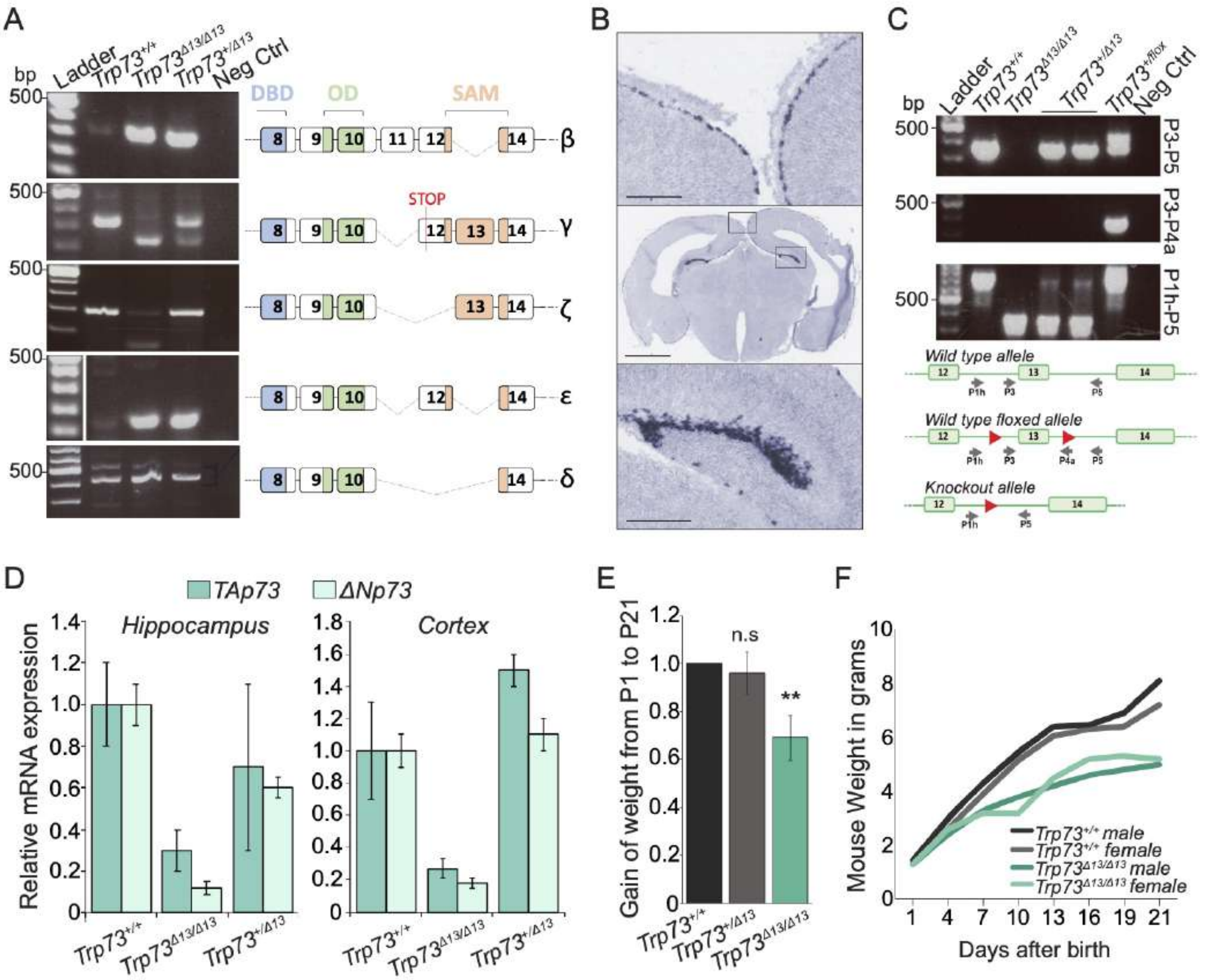

A
bp
Ladder
Trp73+/+
Trp73Δ13/Δ13
Trp73+/Δ13
Neg Ctrl
500
DBD
OD
SAM
STOP
8 9 10 11 12 14 β
8 9 10 12 13 14 γ
8 9 10 13 14 ζ
8 9 10 12 14 ε
8 9 10 14 δ
B
C
bp
Ladder
Trp73+/+
Trp73Δ13/Δ13
Trp73+/Δ13
Trp73+/flox
Neg Ctrl
500
P3-P5
P3-P4a
P1h-P5
Wild type allele
Wild type floxed allele
Knockout allele
12
13
14
P1h
P3
P4a
P5
D
TAp73
ΔNp73
Hippocampus
Cortex
Relative mRNA expression
Trp73+/+
Trp73Δ13/Δ13
Trp73+/Δ13
E
Gain of weight from P1 to P21
n.s
**
Trp73+/+
Trp73+/Δ13
Trp73Δ13/Δ13
F
Mouse Weight in grams
Days after birth
Trp73+/+ male
Trp73+/+ female
Trp73Δ13/Δ13 male
Trp73Δ13/Δ13 female

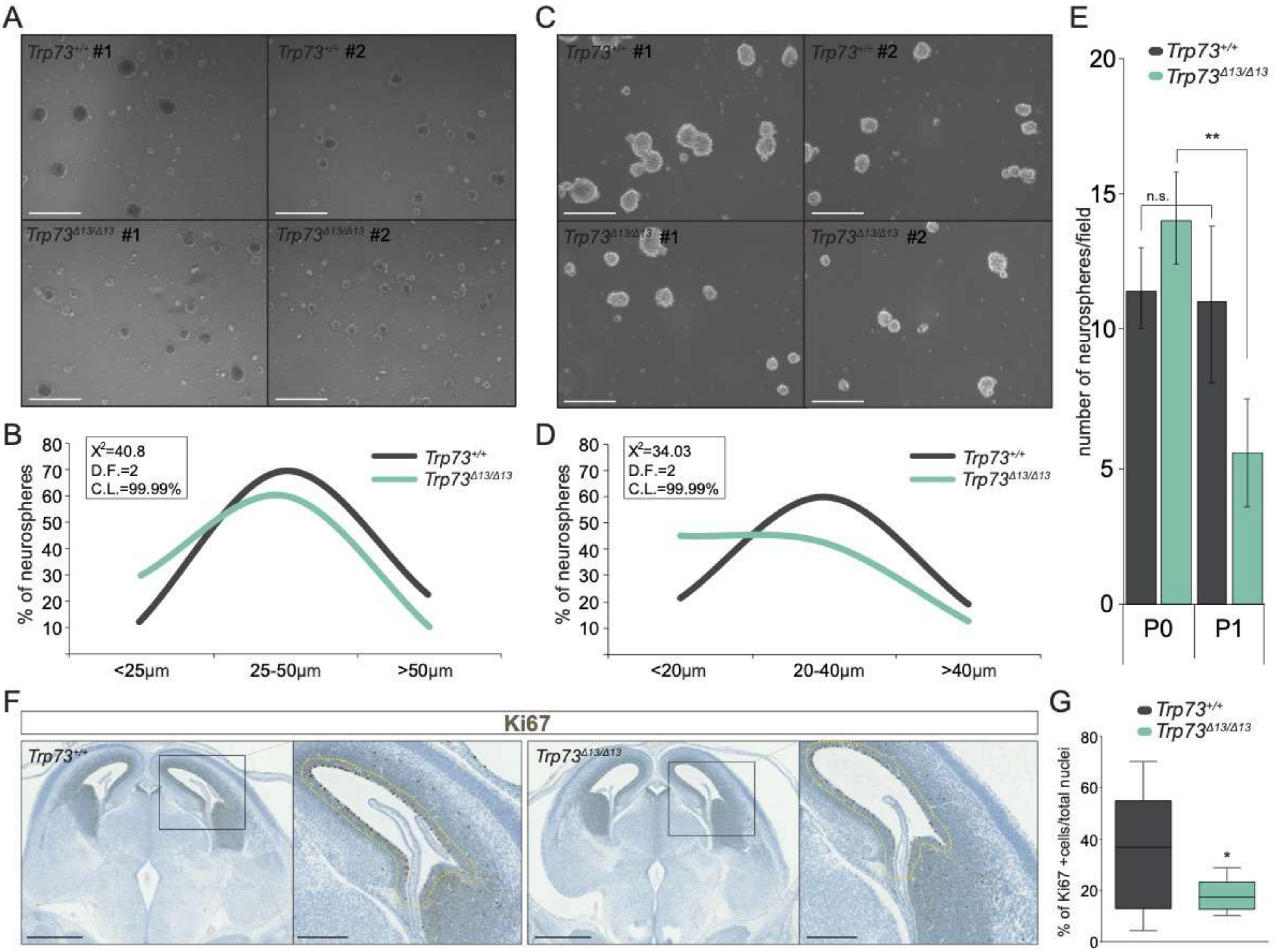

A
Trp73+/+ #1
Trp73+/+ #2
Trp73Δ13/Δ13 #1
Trp73Δ13/Δ13 #2
B
X2=40.8
D.F.=2
C.L.=99.99%
% of neurospheres
<25μm
25-50μm
>50μm
C
D
X2=34.03
<20μm
20-40μm
>40μm
E
number of neurospheres/field
n.s.
**
P0
P1
F
Ki67
G
% of Ki67 +cells/total nuclei
*

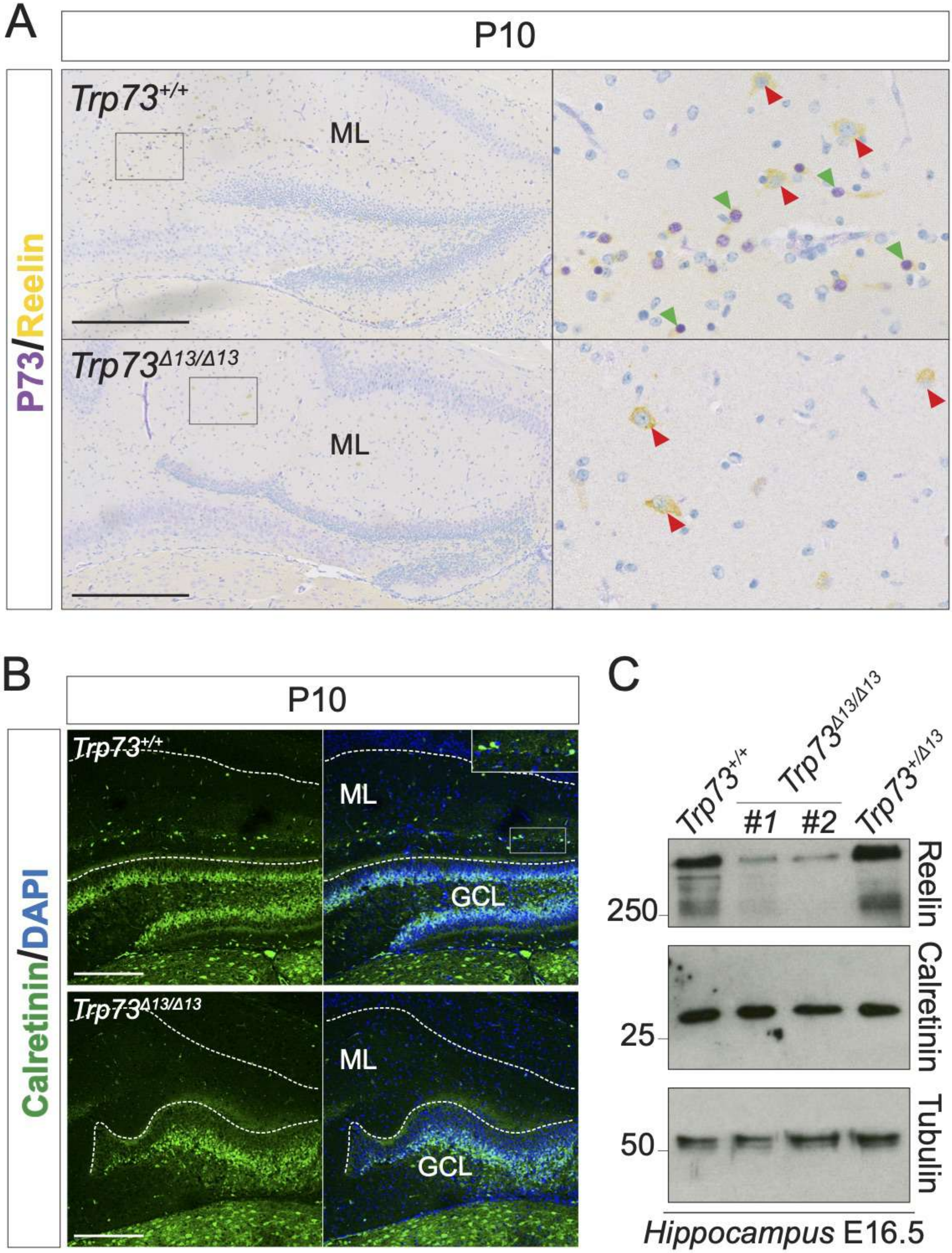
A
P10
Trp73+/+
ML
Trp73Δ13/Δ13
ML
P73/Reelin
B
P10
Trp73+/+
ML
GCL
Trp73Δ13/Δ13
ML
GCL
Calretinin/DAPI
C
Trp73+/+
Trp73Δ13/Δ13
#1
#2
Trp73+/Δ13
Reelin
250
Calretinin
25
Tubulin
50
Hippocampus E16.5

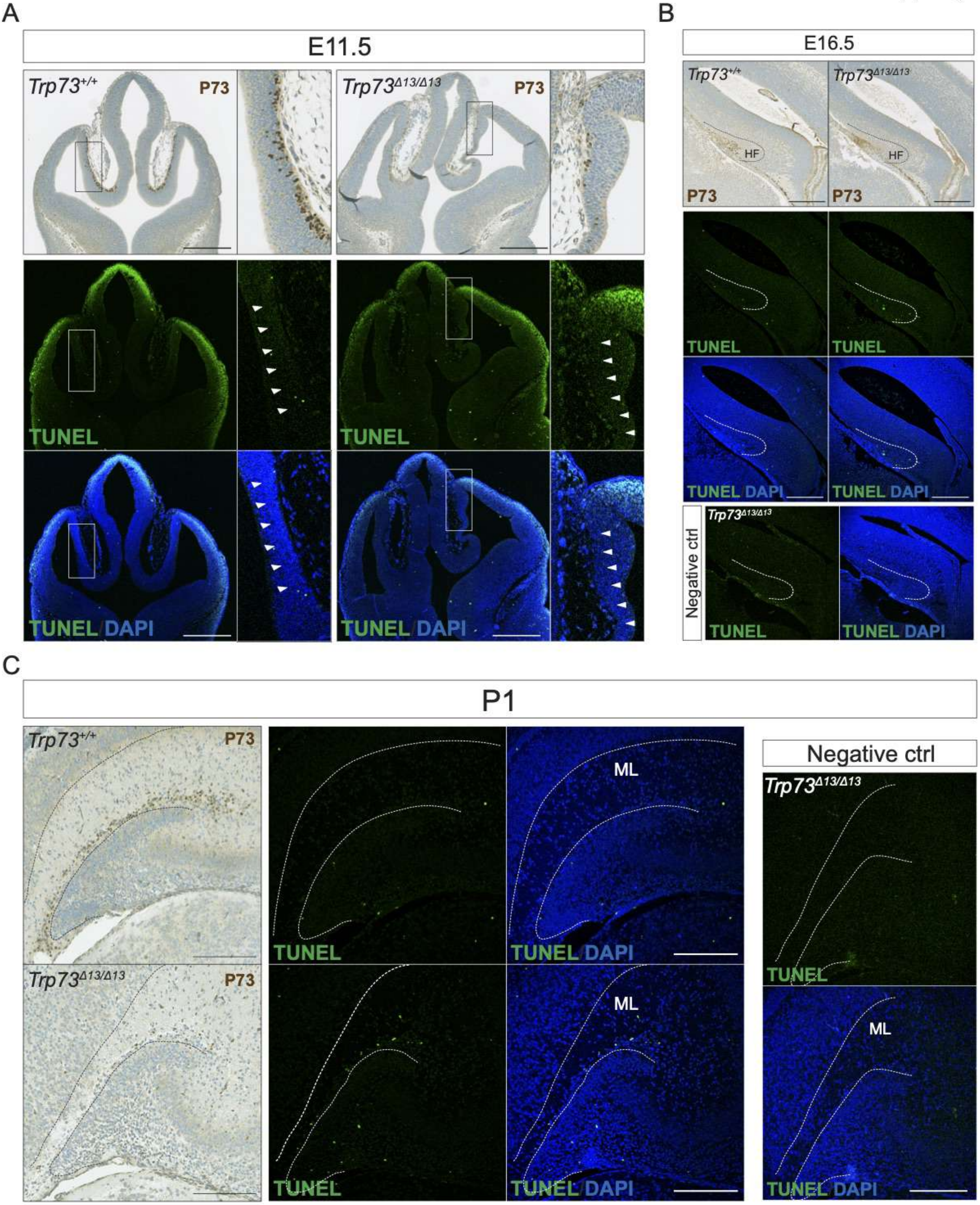
A
E11.5
Trp73+/+
P73
Trp73Δ13/Δ13
P73
TUNEL
TUNEL
TUNEL DAPI
TUNEL DAPI
B
E16.5
Trp73+/+
Trp73Δ13/Δ13
HF
HF
P73
P73
TUNEL
TUNEL
TUNEL DAPI
TUNEL DAPI
Negative ctrl
Trp73Δ13/Δ13
TUNEL
TUNEL DAPI
C
P1
Trp73+/+
P73
TUNEL
ML
TUNEL DAPI
Trp73Δ13/Δ13
P73
TUNEL
ML
TUNEL DAPI
Negative ctrl
Trp73Δ13/Δ13
TUNEL
ML
TUNEL DAPI
